# The Efficacy of a Resilience-Enhancement Program for Mothers Based on Emotion Regulation: A Randomized Controlled Trial in Japan

**DOI:** 10.3390/ijerph192214953

**Published:** 2022-11-13

**Authors:** Hiromi Tobe, Mariko Sakka, Sachiko Kita, Mari Ikeda, Kiyoko Kamibeppu

**Affiliations:** 1 Department of Family Nursing, Global Nursing Research Center, Graduate School of Medicine, The University of Tokyo, 7-3-1 Hongo, Bunkyo-ku 113-0033, Tokyo, Japan; 2 The Faculty of Medicine, University of Tsukuba, 1-1-1 Tennoudai, Tsukuba-shi 305-8575, Ibaraki, Japan; 3 Department of Nursing, Graduate School of Health and Welfare Science, International University of Health and Welfare, 4-1-26 Akasaka, Minato City 107-8402, Tokyo, Japan

**Keywords:** child maltreatment, community-based participatory research, emotion regulation, parents, resilience

## Abstract

This study evaluated the efficacy of a brief (four 2-h sessions) group-based resilience-enhancement program focused on emotion regulation in Japan. Mothers (n = 123) of children aged 3–6 years were recruited in two prefectures and allocated with stratified randomization by the prefecture to either a bi-weekly intervention or treatment as usual. Mothers self-reported online at pre/post-intervention and at 2-month follow-up. Analysis of covariance was used to compare groups. At post-intervention and 2-month follow-up, the intervention group mothers showed significant improvements compared to the control group in resilience (*p* < 0.001/*p* = 0.001), self-esteem (*p* = 0.008/*p* = 0.001), anger control toward the child (*p* < 0.001/*p* = 0.012), and positive attribution toward the child’s misbehavior (*p* < 0.001/*p* = 0.003). The partners of mothers in both groups answered the same questionnaire at the same timepoints without participating in either program; no differences between groups were found. This study was the first randomized controlled trial investigating how a resilience-enhancement program improves maternal resilience, emotion regulation, and cognition toward children and themselves. This preliminary study provides evidence that improving resilience may reduce the risk of child maltreatment. Further research regarding implementing this intervention in the community is warranted.

## 1. Introduction

Resilience has traditionally referred to “the process of, capacity for, or outcome of successful adaptation despite challenging or threatening circumstances” [1]. The initial focus of resilience research has been on traumatic experiences of underserved children; however, resilience has been widely researched over the last 40 years, with resilience studies expanding to encompass the systemic level [2,3,4]. Research has defined resilience multi-dimensionally as a capacity, process, or result of overcoming adversity [5]. A focus on “the capacity of a dynamic system to adapt successfully” reveals themes such as rising above, adaptation and adjustment, and dynamic processes [6,7,8]. Life’s most challenging events and tasks are experienced in the family, and understanding and helping families promote positive adaptation to life’s inevitable challenges is of major importance [9,10].

This is especially true for families with young children as they face day-to-day distress. The parent-child relationship differs from other interpersonal relationships in that it is supported by close ties, such as a blood relationship and parenting responsibility. Due to this deep relationship, various emotions are experienced in child-rearing situations [11]. Moreover, both abusive and non-abusive parents experience parenting stress because they acknowledge the importance of their parental role and try to meet the demands of this role by using their psychological, social, physical, and physiological resources [12,13,14]. Shimizu et al. [15] reported that the emotion that mothers felt the most when experiencing parenting stress was anger. Because parental anger has been associated with child noncompliance, child abuse, dysfunctional discipline strategies, and various emotional problems during childhood, it is of special concern [16]. Maternal irritability and anger were associated with depression and harsh discipline practices, which negatively affect the child’s well-being, even in children as young as 6months of age [17,18,19,20]. Furthermore, alterations in children’s brain structure that increase the risk of psychopathological issues have been linked to verbal aggression from the parents [21,22]. While such deleterious parental behaviors have been associated with problematic adolescent conduct and depressive symptoms, longitudinal and cross-sectional investigations of the role of parental warmth demonstrate mixed associations [4,23,24]. Furthermore, among general communities, many parents are unaware of the negative effect of their harsh verbal and physical disciplining. Parents have anger outbursts and experience regret afterward, which decreases their self-esteem and self-efficacy. Therefore, there is an urgent need to support parents in addressing negative emotions such as anger in order to help prevent child maltreatment and improve their own mental health. 

As parenting stressors are multi-factorial, resilience enhancement against stress is one of the key approaches in addressing related concerns [25,26]. While research on parental resilience has been neglected [27,28,29], the future is bright for resilience research and practices within families and other systems that support human adaptation [30]. Several previous studies have shown the positive effects of resilience enhancement programs in parents of children with serious illnesses [31,32,33] or developmental disabilities [34]. Alongside building resilience against traumatic or serious events, it is also important to consider resilience for day-to-day stressors, especially for younger adults [35,36,37,38]. Beyond addressing maladjustment, building resilience through a parenting intervention can emphasize prevention [39,40]. Promising results affirm that proactive parenting strategies including mindfulness and self-regulation improve resilience and self-confidence for parents and children [41,42].

Emotion regulation (ER) is an important factor of resilience [43,44]. ER refers to the “implementation of a conscious or non-conscious goal to start, stop or otherwise modulate the trajectory of an emotion” to accomplish one’s goal through practical strategies including reappraising the emotional trigger, using prospective taking or self-distanced reflection [45,46,47,48]. ER plays a vital role in the family and is one of the adaptative systems at the forefront of the Family Resilience Model [49,50]. ER is related to family bonding, attachment, communication patterns, parenting style [5,51], and the well-being of children and couples [52]. Parental ER critically affects children’s ER and can cause an intergenerational transmission of emotional response characteristics [53,54,55,56]. In adversity, ineffective ER can increase family tension and disharmony [49]. It is therefore critical for parents to learn effective ER to be resilient and proactive in coaching children to adapt successfully during the turmoil of life. 

Although it is expected that emotional reactions result from stressful events, it is certainly the person’s cognitive appraisal of the situation that triggers the emotional response. Therefore, positive cognitive reappraisal (PCR) aims to reduce negative emotional experiences by positively reframing the perception of the situation [27,57]. PCR and resilience are positively related [58,59,60], while other disturbed or negative ER strategies (e.g., emotion suppression, distraction, blaming others) are negatively associated with well-being [61,62,63,64]. In their comprehensive systematic review on PCR and resilience, Riepenhausen et al. [58] reported that ER strategies based on PCR may be protective to well-being and functioning, improve a person’s ability to cope with stress, and trigger other positive outcomes in healthy participants. 

Maternal stressors are multifaceted and complex [65], and can include children’s misbehavior; lack of social support from a partner or the community; difficulty in relationships with a partner, parents, or co-workers; and low self-esteem or self-image. However, as described above, the perception of such challenging events or situations can be altered by effective training in ER or PCR to build resilience. Mothers may be prone to anger if they hold their children to irrational standards or if they use harsh disciplinary practices. Because resilience strategies are also multifaceted, strengthening multiple protective factors by targeting those that are most modifiable and relevant to the population is possible and encouraged [65,66,67,68,69]. Such factors include skills in ER, stress coping, self-esteem, effective problem solving, and community building through peer and family relationships [70,71]. Parents can accept their imperfections and weaknesses to enhance resilience by building a positive self-image [72,73]. Implementing PCR principles in response to stressful situations should improve ER, build resilience, and may decrease parental anger and anger expression. However, studies investigating the efficacy of such a resilience-enhancement program among mothers have not been reported. The aim of the current study was to investigate the efficacy of a brief, group-based, resilience-enhancement program for mothers, called the *Family Resilience Program* (FRP), focused on ER with a randomized controlled trial in Japan. 

## 2. Materials and Methods

### 2.1. The Current Study

The FRP of the current study was born of a community-based participatory research (CBPR) project. Some parent-support group members in Shizuoka Prefecture, Japan initiated the project by contacting the primary researcher regarding their previous research on an anger management program for mothers [74]. The group members were involved in a project to make suggestions to improve the social environment in their city based on a survey they had conducted among mothers with preschool-aged children. The survey results suggested that despite the increased parent- support services and resources in their city, parenting stress was high. This led them to seek a program that would provide internal support (such as psychoeducation) to improve parents’ cognitive appraisal alongside the external support already present in their community. Through much discussion between the researchers and community members, cognitive interviews with some parents of young children, and consulting experts in the field of family nursing and family therapy, the resilience-enhancement program, FRP, was developed. The conceptual framework was based on cognitive behavior theory (CBT) and the resilience theory and the intervention was developed with the aim of making it user-friendly for the facilitators and participants. The intervention is a brief, low-burden program consisting of four sessions delivered bi-weekly to be applied by health professionals, including public health nurses, who are the gatekeepers for preventing adverse health and well-being outcomes among community members in Japan. 

FRP leveraged core-resilience-building strategies [75,76], including ER, proactivity and optimism, realistic cognitive appraisal, a solution-focused approach, self-esteem, and self-efficacy. Specifically, FRP seeks to support participants using PCT strategies to enhance their skills in the following areas: (1) general stressful life-events—to improve resilience, trait anger, and solution-focused coping; (2) relationship with children—to improve cognition on children’s misbehavior and parental emotion, leading to better anger control in interactions with children; (3) relationship with partners—to improve marital communication and compassion toward/from a partner, leading to better anger control in interactions with partners; and (4) relationship with themselves—to improve self-esteem, which also leads to resilience. 

To evaluate the efficacy of the above FRP, this study investigated the following hypotheses: 

Compared with the control group post-intervention and at 2-month follow-up, in the intervention group,

1. Maternal resilience will be significantly higher.
2. Maternal trait anger will be significantly lower and self-esteem, family functioning, and problem-focused coping will be significantly higher.
3. Maternal anger toward children will be significantly lower and anger control toward children, cognition of children’s misbehavior, and parenting emotions will be significantly better.
4. Maternal anger toward a partner will be significantly lower and anger control toward a partner and marital communication will be significantly higher.
5. The paternal outcomes above will not be significantly different.

In this study, only mothers participated in the program, but they were given homework assignments to share what they learned with their partners. Their partners were asked to answer the same questionnaires to investigate any indirect influences the program may have due to communication or changes experienced by the mothers. We hypothesized that this indirect influence would not be as effective as participation in the program.

### 2.2. Study Design

A randomized controlled trial was conducted for mothers in the Shizuoka and Ibaraki prefectures in Japan from January to October 2017.

### 2.3. Participants 

Mothers aged over 18 years were potential participants for this study. Inclusion criteria were as follows: (a) having sufficient Japanese speaking ability, (b) having and living with at least one child aged 3–6 years, (c) having a partner who could answer questionnaires, and (d) being available to participate in four bi-weekly programs. Mothers who exhibited mental health problems were excluded from this study. 

### 2.4. Procedure 

Eligible mothers were recruited in the kindergartens and nursery schools in the two prefectures in Japan by the researcher (HT) and two research assistants with the aid of posters and fliers. If mothers were interested in this study, they were asked to contact the researcher by phone or email address, which was provided on the flyer. When the researcher received the phone call or email, they provided a detailed explanation of this study (e.g., study design, procedure, and ethical considerations) to the mother. Once mothers agreed to participate, they were sent the consent form and asked to return the signed form to the researcher. After the researcher confirmed their consent, they were immediately and randomly allocated into one of the two groups (i.e., intervention and control) by a research assistant who was not involved in the intervention and analysis. After the randomization, they were sent the questionnaire before the intervention and provided the schedule of the intervention or usual care (small group discussion over coffee) by email. Mothers in the intervention and control groups were provided four sessions of the resilience program and usual care, respectively. When necessary, a babysitting service, housed in a different room in the same building, was available to participants of both groups upon request. Immediately after and 2 months after completing the intervention or usual care, the mothers were provided the questionnaire by email and asked to complete it online. 

### 2.5. Power Calculation

Because of the lack of similar work in Japan, power calculations were exploratory. We calculated that 64 parent-child dyads were needed in each group to detect an effect size of d = 0.62 with a two-tailed α = 0.05 (two-tailed) and β = 0.8. We recruited 140 dyads after considering the possibility of attrition.

### 2.6. Randomization

The randomization in this non-blinded study was stratified by the participants’ home location and was performed by unaffiliated researchers. After baseline data collection, participants were randomly assigned to the intervention or the control group evenly. After the 2-month follow-up data collection process, the control group participants received the intervention. 

### 2.7. Ethical Procedures

Study protocols were approved by the Institutional Review Board of The University of Tokyo Graduate School of Medicine (Approval No.: 11444) and were registered (UMIN000027232). Only consenting participants were included and no incentives were given except for refreshments provided during the sessions. Participants were forewarned that if severe abusive situations became apparent, researchers would report them to the local health services, but no such action was required. 

### 2.8. Intervention Group

In the intervention group, participants were divided into groups of five to 11 participants according to their schedule availability. They attended four bi-weekly 2-h sessions facilitated by a researcher (HT). Various methods were used including psychoeducation, interactive didactics (interactive discussion regarding the aforementioned resilience protective factors), and behavioral rehearsal (e.g., role-plays, positive practice, experiential exercise). Between-session assignments (e.g., monitoring and recording) were provided and sharing time was set in the beginning of each session as an essential part to practice applying the skills in their daily lives and to learn from each other. In the case of absence, participants could join the same session with the other group. Alternatively, the researcher sent them the material, called them and briefly described the lesson content, and invited them to share their experience and answer their questions. A detailed description of the session content was published previously [77]; a brief description is provided in Table 1. 

### 2.9. Control Group

Mothers in the control group were clustered with five to 10 members for discussion over coffee based on their schedule availability. As treatment as usual, they freely discussed the stressful events experienced with their children, partners, and others and how they managed them. Research assistants supported their discussion, asking participants questions based on the content of each session in the intervention group. However, they did not teach or support participants to join the discussion.

### 2.10. Measures

Mothers and their partners completed the set of questionnaires described below at baseline (T0), post-intervention (immediately after) (T1), and at 2-month follow-up (T2).

#### 2.10.1. Demographic Information

We collected data on participants’ age, gender, education level, employment and marital statuses and household income. With regard to the children, data were collected on the age of the oldest child and the number of children. 

#### 2.10.2. Primary Outcome

The primary outcome was the self-report assessments of resilience at T2. We used the Psychological Resilience Scale [78] originally developed in Japan to measure the level of resilience with reportedly high construct validity. This scale consists of 21 items scored on a five-point scale and three subscales. Examples include “I’m curious about many things” (novelty pursuit); “I can control my anger” (ER); and “I believe I have a bright future” (positive future outlook). Higher scores indicate a higher degree of resilience. The Cronbach α in this study was 0.83–0.89. 

#### 2.10.3. Secondary Outcomes

State-trait anger expression inventory (STAXI): We used the Japanese version of the STAXI [79,80], which measures five components of anger. This reliable and valid measure is widely used. The five subscales include state anger (how you currently feel), trait anger (how you typically feel), anger-in (anger suppression), anger-out (anger expression), and anger control (anger control or appropriateness of expression). We assessed anger toward children and partners independently using the five components because anger may be felt differently in different contexts. The Cronbach α in this study was 0.78–0.89. 

Self-esteem: We measured self-esteem with the Japanese version of the Rosenberg’s Self-Esteem Scale that consists of 10 items, with high scores indicating more positive self-esteem [81]. Its reliability and validity were identified. The Cronbach α was 0.87–0.89. 

Family functioning: We used the Japanese version of the Family APGAR which measures five parameters of family functioning including adaptability, partnership, growth, affection, and resolve [82]. Its reliability and validity were identified [83]. Higher scores indicate better family functioning. The Cronbach α was 0.82–0.88. 

Cognition toward child’s behavior: the Scale of Mother’s Recognitions on Child’s Behaviors was developed by Nakaya and Nakaya [11]. It has three subscales: hostile, negative, and positive attribution toward a child’s misbehavior. Hostile items include “I feel I am being denied,” and “I feel I am being ignored”; negative items include “I don’t know what to do.” Positive items include “I feel my child is growing.” Higher scores for each subscale indicate higher degrees of the mothers’ hostile, negative, or positive attributions to their children’s behaviors. This scale has been reported to be reliable and significantly correlated with scores on the Self-Esteem Scale. The Cronbach’s α of the scale in this study was 0.84–0.89.

Parenting emotion: The Parenting Emotion Scale was developed in Japan and consists of five subscales measuring four negative and one positive attitude [84]. Examples include “I can’t do what I want to do, I’m spending too much time on parenting” (burden from parenting), “I feel disgusted when children make a mess” (burden from children’s attitude and behavior), “I’m not confident about my parenting” (anxiety about parenting), “I’m afraid my child is more childish than others” (anxiety about child’s development), and “I feel I’m growing through parenting” (positive parenting attitude). Higher scores indicate a higher degree of each attitude. The Cronbach α in this study was 0.70–0.89. 

Marital communication attitude: To assess the mode of communication between husband and wife, we used the Marital Communication Attitude Scale [85]. It considers the husband and wife as individuals who constitute communication and asks both the husband and wife to answer questions about the communication style between themselves and the other party. It consists of four subscales: intimidation, empathy, dependence and approach, and ignorance and avoidance. The Cronbach α for each subscale is 0.69–0.79. The higher the score of each scale, the more it indicates that the participant possesses or experiences the particular aspect. In this study, we used a subscale of empathetic attitudes (e.g., I say [My partner says] kind words when my partner is [I am] not feeling well) because the FRP program included content that encouraged empathetic attitudes. The Cronbach α in this study was 0.79–0.88. 

Problem-focused coping strategies: To measure how well the parents cope with stress, we used The Problem-Focused Coping Strategies Scale, which has been shown to be reliable and valid [86]. This scale includes five subscales; in this study, we used the solution calculation and the concrete solution behavior measures which best matched the purposes of the program. The Cronbach α was 0.77–0.89. 

### 2.11. Statistical Analysis 

We used the statistical package IBM SPSS Statistics software (SPSS) version 29 (SPSS Inc., Chicago, IL, USA, USQ) to calculate the Welch’s *t*-test or Fisher’s exact tests to descriptively compare the group baseline characteristics. We used a student’s *t*-test to compare the group baseline measures. An analysis of covariance (ANCOVA) was used with baseline as a covariate to see the statistical difference between the groups at post intervention and at 2-month follow-up. A repeated measures analysis of variance (ANOVA) was used to evaluate the Group by Time effect from baseline through 2-month follow-up. The significance level was set at *p* < 0.05.

## 3. Results

### 3.1. Flow of Participants

A total of 147 mothers contacted the researcher with interest in the study. Among them, 24 mothers did not engage in the study due to scheduling conflicts (n = 16) or unwillingness to participate (n = 8). Thus, 123 mothers agreed to participate and were randomly allocated into the two group: the intervention group (n = 62) and the control group (n = 61). In the intervention group, 62 mothers and 55 partners completed the questionnaire pre intervention and 56 mothers received the intervention. After that, 52 mothers and 39 partners answered the questionnaires post intervention and 42 mothers and 24 partners answered them at the 2-month follow-up. Data from the 52 mothers and 39 partners were used for the post-intervention analysis right after the intervention and data from 42 mothers and 24 partners were used for the analysis at the 2-month follow-up.

**Figure 1:**
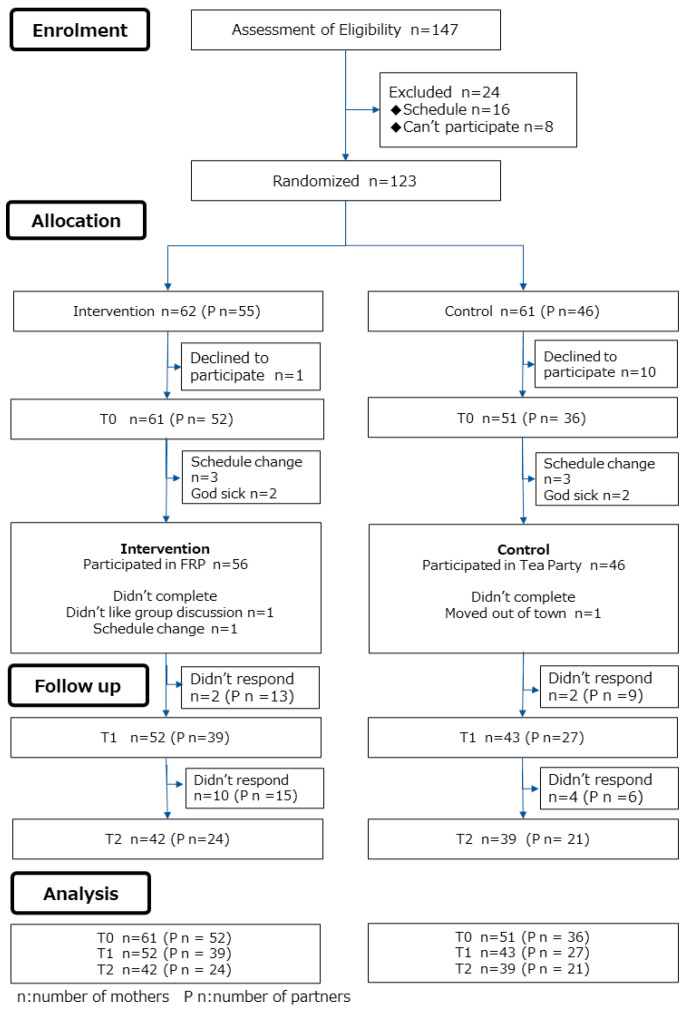
Flowchart.

In the control group, 61 mothers and 46 partners completed the questionnaire before the intervention and 51 mothers attended group discussions. After that, 46 mothers and 27 partners answered the questionnaires right after the intervention and 39 mothers and 21 partners at the 2-month follow-up. Data from 43 mothers and 27 partners were used for the analysis right after the intervention and data from 39 mothers and 21 partners were used for the analysis at 2-month follow-up (Figure 1).

### 3.2. Demographics and Baseline Data of the Participants 

The mean age of the mothers, fathers, and children was 40, 42, and 5.6, respectively, in both the intervention and control groups (Table 2). The average number of children was two. Approximately 60% of the mothers were not working. Welch’s *t*-test or Fisher’s exact tests found no differences between the intervention and control groups for all demographics at baseline, except for the father’s educational background (*p* = 0.017), as presented in Table 3. Approximately half of the mothers and more than 70% of the partners graduated from university or graduate school. Only the educational background of partners was significantly different between the intervention and control groups: university = 64.2 (intervention) vs. 47.2 (control). Concerning the mean score of resilience regarding the baseline data, none of the scores of the main variables was statistically different between the intervention and control groups.

### 3.3. Effect on Primary Outcome

The total score of maternal resilience at 2-month follow-up, which was our primary outcome, was significantly higher among the intervention group compared to the control group (*F* = 10.934, *p* = 0.001) (Table 3).

### 3.4. Effects on the Secondary Outcomes

#### 3.4.1. Effects on the Secondary Outcomes (Mothers)

The total score of maternal resilience post intervention in the intervention group was significantly higher than the control group (*F* = 14.257, *p* < 0.001). In addition, among the subscales of maternal resilience, there was a significant difference between groups post intervention and 2-month follow-up in emotion regulation (*F* = 7.634, *p* = 0.007)/(*F* = 17.905, *p* < 0.001) and future outlook (*F* = 14.860, *p* < 0.001)/(*F* = 8.572, *p* = 0.004). There was no significant difference between the groups in novelty seeking (Table 3). 

The score of trait anger in the intervention group post intervention (*F* = 7.153, *p* = 0.009) was significantly lower than the control group. The anger-out toward children score was significantly lower both post intervention (*F* =6.942, *p* = 0.010) and at 2-month follow-up (*F* = 4.329, *p* = 0.041) and anger-control toward children score was significantly higher both post intervention (*F* = 14.122, *p* < 0.001) and at 2-month follow-up (*F* = 6.643, *p* = 0.012) among the intervention compared to the control group. The anger-out toward partner score was significantly lower post intervention (*F* = 8.875, *p* = 0.004) and the anger-control toward partner score was significantly higher both post intervention (*F* = 4.628, *p* = 0.034) and at 2-month follow-up (*F* = 6.193, *p* = 0.015) among the intervention compared to the control group. There was no significant difference between the groups in anger-in toward both children and partner.

The self-esteem scores in the intervention group at post intervention (*F* = 7.389, *p* = 0.008) and 2-month follow-up (*F* = 18.767, *p* < 0.001) were significantly higher than the control group. 

The family function scores in the intervention group at post-intervention (*F* = 7.968, *p* = 0.006) and 2-month follow-up (*F* = 3.996, *p* = 0.049) were significantly higher than the control group.

For cognition toward child’s misbehavior, the scores of hostile attribution (*F* = 6.945, *p* = 0.010) and negative attribution (*F* = 7.121, *p* = 0.009) at 2-month follow-up were significantly lower, and positive attribution both at post intervention (*F* = 16.636, *p* < 0.001) and 2-month follow-up (*F* = 9.227, *p* = 0.003) were significantly higher in the intervention than the control group. 

For parenting emotion, among subscales in negative emotions, burden from parenting (*F* = 8.956, *p* = 0.004) and anxiety about parenting (*F* = 11.606, *p* < 0.001) post intervention, burden from child’s attitude/behavior score (*F* = 22.098, *p* < 0.001/*F* = 9.237, *p* = 0.003) and anxiety about child’s development score (*F* = 7.567, *p* = 0.007/*F* = 6.346, *p* = 0.014) both at post intervention and 2-month follow-up were significantly lower and positive emotion about parenting score (*F* = 4.413, *p* = 0.038/*F* = 5.541, *p* = 0.022) both at post intervention and 2-month follow-up were significantly higher in the control group than the control group. 

For marital communication attitude, compassion toward partner score post intervention (*F* = 4.060, *p* = 0.047) and compassion from partner score at 2-month follow-up (*F* = 5.389, *p* = 0.023) were significantly higher than the control group. 

For problem-focused coping, solution seeking score (*F* = 15.638, *p* < 0.001/*F* = 6.061, *p* = 0.016) and solution behavior score (*F* = 11.918, *p* = 0.001/*F* = 6.435, *p* = 0.013) in the intervention group were significantly higher than the control group post intervention and at 2-month follow-up.

The group by time effect showed that there was a significant difference between the groups, which indicates improvement in resilience (*F* = 9.552, *p* < 0.001), emotion regulation (*F* = 10.540, *p* < 0.001), future outlook (*F* = 7.383, *p* = 0.001), novelty seeking (*F* = 3.734, *p* = 0.028), trait anger (*F* = 4.145, *p* = 0.019), anger-out toward children (*F* = 4.697, *p* = 0.012), anger-control toward children (*F* = 3.205, *p* = 0.046), anger-out toward partner (*F* = 4.326, *p* = 0.017), anger-control toward partner (*F* = 4.935, *p* = 0.016), self-esteem (*F* = 9.788, *p* < 0.001), family functioning (*F* = 3.679, *p* = 0.030), negative attribution toward child’s misbehavior (*F* = 3.828, *p* = 0.026), positive attribution toward child’s misbehavior (*F* = 5.066, *p* = 0.009), burden from parenting (*F* = 6.770, *p* = 0.002), burden from child’s attitude/behavior (*F* = 14.008, *p* < 0.001), anxiety about parenting (*F* = 7.890, *p* < 0.001), anxiety about child’s development (*F* = 5.027, *p* = 0.009), positive emotion about parenting (*F* = 4.015, *p* = 0.022), solution seeking (*F* = 5.426, *p* = 0.006), and solution behavior (*F* = 6.731, *p* = 0.002).

#### 3.4.2. Effects on the Secondary Outcomes (Fathers)

None of the variables of partners showed a significant difference between the intervention and control group both at post intervention and 2-month follow-up (Table 4).

### 3.5. Acceptability and Feasibility

The majority of mothers in the intervention group reported to have shared 20–30% or 50% of what they learned in the program with their partners (48.1% and 25.0%, respectively).

Furthermore, 86% and 13.5% of mothers reported to be very satisfied and satisfied with the intervention, respectively. Of the 54 mothers who attended the intervention, 90.7% completed all four sessions and 9.3% completed three sessions.

## 4. Discussion

To the best of our knowledge, this was the first randomized controlled trial to evaluate the efficacy of a resilience-enhancement program for mothers focused on ER in Japan. This study’s findings suggest that the FRP had a positive effect on improving mothers’ resilience, trait anger, anger expression, cognition on children’s misbehavior, parenting emotions, self-esteem, marital communication, and solution-focused coping. The originality of this research was that the intervention addressed not only parenting-related stress, but also general stress and partner or self-related stress using a CBT approach to enhance resilience, a strength-based concept. This study provides an important contribution to the literature on family resilience and ER. 

As hypothesized, the primary outcome of maternal resilience significantly improved in the intervention group. This was also the case for trait anger, self-esteem, family functioning, and problem-focused coping. ER was the focal point of the intervention because high ER has been associated with high resilience [43,44,87,88,89,90,91]. Through the FRP, it is possible that mothers gained various knowledge and skills, including PCR; attention control; and appropriate expression of stress emotions, including anger, that enabled them to regulate their emotions, which may have affected their resilience. ER also directly affects communication patterns and the parent-child and marital relationship [51,92], as observed in the improved family functioning results in this study, especially related to maternal anger [93]. Previous research has shown that when stressful events occur, if mothers react angrily, it further heightens family tension and vulnerability [51]. Therefore, the FRP taught mothers that cognitive reappraisal is key for improving their ER and resilience that can benefit their relationships with their children, partners, and themselves. As protective factors of resilience are interdependent and interrelated, improvement in each factor may have enhanced multiple other factors along with resilience [75]. 

Regarding child-related outcomes, the FRP showed significant positive results in anger-out and anger control, with no significant difference in anger-in toward children post intervention and at 2-month follow-up, as hypothesized. Considering that trait-anger was significantly lower post intervention, this result indicates that the FRP was effective in teaching mothers that anger is a natural emotion and that suppressing it has a negative impact on physical and mental health. The mothers also learned that directing their anger inappropriately at children has negative short- and long-term effects, and they practiced assertive communication skills which may have contributed to their improved anger expression. 

The mothers’ cognition with regard to their child’s misbehavior and their parenting emotions also significantly improved. Previous research shows that mothers are more likely to feel angry toward their children’s disobedient attitudes and behaviors [94], and that anger is associated with intensive parenting attitudes and ER [69]. Through the FRP, the mothers learned to understand child development more fully, which might have led to more realistic expectations and less hostile (e.g., “My child is doing it on purpose”), less negative (e.g., “I don’t know what to do”), and more positive (e.g., “My child is growing”) attribution. Knowing and practicing more appropriate responses toward children’s misbehavior may have also led to less negative parenting emotions (e.g., burden from parenting). Hostile attribution toward children’s misbehavior has been associated with anger [11]. Nakaya et al. [95] stated that if we can encourage the modification or transformation of these attributions, it may reduce the associated negative feelings and improve inappropriate nurturing. Bugental et al. [96] further reasoned that providing careful guidance about the child’s development, staying with the child until a realistic interpretation of the child’s misbehavior is possible, and fostering parents’ experiences of success in difficult situations can also contribute to reducing frustration. 

In the FRP, we promoted better understanding of children’s misbehaviors by providing specific potential responses for the mothers; we asked each mother to report on how they had practiced them and acknowledged even the small progress made. We likened the practice of the new skills they learned in the program to learning a new foreign language and emphasized the importance of practicing despite difficulties. We believe that reinforcing small accomplishments may have contributed to the mothers’ cognitive transformation. The mothers’ overall change in cognition toward their children may have influenced their parenting emotions and perceived burden and anxiety. Improvement in the hostile attribution likely contributed to a decrease in anger [11,95] and less negative cognition to a decrease in negative parenting emotions. Nurturing the parents’ experience of success in difficult situations also contributes to reducing frustration [95] and may have contributed to the successful results we observed. 

Regarding partner-related outcomes, the FRP group improved and maintained anger control toward their partner and compassion from their partner at both timepoints, while they improved anger-out and compassion toward their partner only post intervention. These results indicate that the FRP to help mothers to use PCR in marital relationships was effective and sustainable at 2-month follow-up; however, the decrease in expressing anger and increase in expressing compassion did not last. During the report and reflection time of each session, many mothers reported that the assignment of building a better relationship with their partners was difficult compared to that with their child. Despite this challenge, it is noteworthy that they recognized the compassion from their partners more. Other studies describe that the partner relationship plays a key role in the situational factors related to parental stress [97], and that mothers’ perception of their husband’s support is a major influence on their parenting stress and that the anger-hostility factor stems partially from a lack of partner support [98]. The empathetic attitude from the husbands in the present study can be viewed as emotional support, and it is possible that anger control was maintained 2 months later because of greater recognition of that emotional support. In Shimizu’s study [15], the most frequently cited content of parenting stress by mothers, next to children’s self-centered characteristics, was their relationship with their husbands, with the following specifics: “lack of cooperation in child rearing,” “housework not done unless told,” “no understanding or words to say that I am raising children,” and “they only live around themselves”; this was also mentioned by mothers in the present study. These specifics suggest that transforming cognition alone does not alleviate partner-related stress and negative emotions, including anger; however, receiving partner support is beneficial for maternal anger management [99]. 

As hypothesized, paternal outcomes in the intervention group were not significantly different from the control group at both timepoints. Several reasons for this need to be considered. First, paternal outcomes might be significantly different from mothers at baseline (paternal resilience might be higher and anger lower at baseline, with no space for improvement). Second, only 25% of the mothers shared the acquired knowledge and learning with their partners, and indirect influences were not shown. Third, limited sharing is not initially effective in changing the outcome. However, indirect influence can be realized not through communication but through observing the change in mothers or the mother-child relationship. 

The current FRP can be considered a feasible psychological educational program for busy mothers with young children in view of the low attrition rate and high attendance rate observed. One benefit of the resilience enhancement approach was that participants were receptive to engage and develop new skills or enhance their strengths [100]. With this approach, programs can harness the mothers’ strengths and new skills to drive positive changes in the future welfare of their children and themselves by letting go of regrets with regard to their past inappropriate parenting [66]. Participating in this approach clearly strengthens resilience [66], which empowers meaningful change. Riepenhausen et al. [58] demonstrated particularly large effects on resilience through training strategies that increase the frequency or success of PCT. Thus, a focus on ER using PCR on mothers’ main stressful events and situations could have been effective in improving and sustaining resilience even at 2-month follow-up. 

Despite these important findings, some limitations should be noted. First, the small sample size and sample bias limit the generalizability of the results to a larger population. Participants were more highly educated and worked less than the average Japanese mothers [101]. They might have had more understanding, motivation, and time for learning and in applying what they learned, which might have affected the results. Some studies showed that non-working mothers were more stressed and likely to get angry compared to working mothers [94,96]. Further research may need a larger sample size to include more variety in educational background and working status.

Secondly, the results are limited with the post-test assessment only 2 months after program delivery and the self-report questionnaire. A longer-term follow-up with multiple observational outcomes, including biological assessment, may clarify the effectiveness of this program more accurately. Third, the assessment sheet did not include any data regarding children, including developmental issues or characteristics, which also affect the level of parenting stress. Further research may need to include these data to ensure that the program can be applied according to the special needs of each family. Fourth, because there was only one facilitator for the program, the efficacy may depend heavily on their skills, experiences, and personality. An important next step may be to train other facilitators in using the program material and validate that the same quality could be delivered in future implementations of the program. 

Another limitation to consider is that the current study included partners only to answer questionnaires to investigate the indirect influences from mothers who participated in the intervention. Fathers are underrepresented in parenting research overall [66], and in the current study, 30% of the partners hoped to participate in a similar program and 40% were interested in the program; this should be noted and addressed in future studies. The birth of a child can be a crisis for couples, as it strengthens the mother-child relationship and changes and challenges the husband-wife partnership [102]. Involving fathers in parenting does not only decrease mothers’ stress, but also promotes and greatly affects children’s and fathers’ development and growth [16,103,104]. Japan is a peculiar country where men are less involved in parenting, which seriously harms the psychological development of children and adults [102]. However, reportedly, fathers are increasingly getting involved in parenting and housework; they are called “Ikumen,” meaning “parenting men” in Japan. Nevertheless, irrespective of such efforts of the fathers, their partners often consider they are not doing enough and show dissatisfaction, which leads to increased stress for fathers. [105]. Considerate assessment of the common and different stress experienced by mothers and fathers should be conducted and an intervention which meets their respective needs developed so that they can be aware of each other’s different needs and utilize each other’s strengths as they co-parent. 

The current study proposes several important public health implications. Alvord et al. [75] suggested that the effectiveness of resilience interventions may be strengthened by involving parent-family and community systems alongside the individual. They also suggested that it would be important for practitioners and researchers to examine and incorporate the cultural context, mediators, and moderatos as well as which components of the interventions have the greatest impact to advance the theoretical understanding of resilience. This CBPR continues to seek ways to apply these findings in the community. Further research is needed to qualitatively investigate the effect and mechanism of the program for improving its effectiveness and sustainability. Preparing simple and efficient training systems and materials should be the next step for application in the service by public health nurses and other professionals. Furthermore, research to explore its effect and feasibility should be conducted. To this end, including fathers would be an important aspect to enhance resilience as a family system.

## 5. Conclusions

The current study investigated the efficacy of a brief, group CBT-based resilience-enhancement program, FRP, focused on ER among Japanese mothers with a randomized controlled trial. The results demonstrate preliminary evidence of the efficacy of the FRP focused on ER using the PCR approach for improving resilience, anger control, cognition on child’s misbehavior, parenting emotions, self-esteem, marital communication, and solution-focused coping. Further research is needed to investigate its effectiveness with a larger sample, including fathers, with longer follow-up with trained health care professionals working in the community as facilitators. Overall, this study proposes an innovative approach by introducing cognitive aspects to advancing evidence-based support for families in the community. 

## Figures and Tables

**Table 1:** Session content.

Session	Learning Point	Theme	Content
1	Resilience, ER, PCR	Every emotion is a dear friend.	We can strengthen parent and child resilience through protective factors of resilience including ER, PCR, assertive communication, positive outlook, self-esteem, self-efficacy, and self-care. Homework: Monitor and modify expression by completing the anger record and appropriate anger scale.
2	Improving the parent-child relationship	Worry less and trust more.	How harsh verbal discipline affects the child’s brain and emotional developmental processes. How to reframe children’s weaknesses as strengths. Homework: Active listening, noticing and praising ordinary appropriate behavior. Showing empathy and validating child’s feelings during problem behavior. Record-keeping encouraged.
3	Improving the partner relationship	Differences are strengths.	How to use PCR to recognize a partner’s differences or weaknesses as strengths. Write five qualities of the partner and five aspects of the relationship they appreciate. Homework: Share what they wrote with partners. Express gratitude and appreciation.
4	Improving the relationship with yourself	I love and trust myself no matter what.	Self-compassion and self-acceptance are best for building resilience and competing with low self-esteem and anger in parents and children. Simple exercises: Look back at lowest moments and identify the strengths gained, write a “thank me letter,” and read to each other. Reflect on past session and plan for the future. Homework: Use self-affirmation more often.

ER: emotion regulation, PCR: positive cognitive reappraisal.

**Table 2:** Demographics.

	Total		Intervention		Control		
	Mothers (n = 112)		Mothers (n = 61)		Mothers (n = 51)		*p* ^a^
	Fathers (n = 88)		Fathers (n = 52)		Fathers (n = 36)		
	*M* (*SD*)/n (%)		*M* (*SD*)/n (%)		*M* (*SD*)/n (%)		
**Age**							
Mothers	40.5	4.87	40.8	4.6	40.1	(5.2)	0.45
Fathers			42.8	5.2	41.1	(5.3)	0.154
Oldest child	5.7	3.1	5.7	2.8	5.6	(3.4)	0.893
**Number of children**	2	0.7	2.0	0.8	2.0	(0.7)	0.808
1	26	23.2	15	24.6	11	21.6	
2	66	58.9	34	55.7	32	62.7	
3	15	13.4	10	16.4	5	9.8	
4	4	3.6	2	3.3	2	3.9	
5	1	0.9	0	0.0	1	2.0	
Education							
Mothers							0.54
High school	13	11.6	9	14.8	4	7.8	
Junior college	38	33.9	21	34.4	17	33.3	
University	49	43.8	25	41.0	24	47.1	
Graduate School	11	9.8	6	9.8	5	9.8	
Fathers							0.017
Junior high school	1	1.1	0	0	1	2.8	
High school	8	9.1	6	11.3	2	5.6	
Junior college	10	11.4	6	11.3	4	11.1	
University	51	58.0	34	64.2	17	47.2	
Graduate School	16	18.2	4	7.5	12	33.3	
No response	3	3.4	3	5.7	0	0.0	
Working status							
Mothers							0.27
Full-time	22	19.6	12	19.7	10	19.6	
Part-time	17	15.2	11	18.0	5	9.8	
Self-employed	4	3.6	2	3.3	2	3.9	
Not working	70	62.5	36	59.0	34	66.7	
Fathers							0.47
Full-time	81	92.0	48	90.6	33	91.7	
Self-employed	5	5.7	2	3.8	3	8.3	
No response	3	3.4	3	5.7	0	0	
Economy (Mother-rated)							0.19
Well-off	9	8.0	4	6.6	5	9.8	
Rather well-off	18	16.1	10	16.4	8	15.7	
Normal	58	51.8	31	50.8	27	52.9	
Rather bad-off	19	17.0	14	23.0	5	9.8	
Bad-off	8	7.1	2	3.3	6	11.8	

^a^: Welche’s *t*-test or Fisher’s exact test; *SD* = Standard deviation.

**Table 3:** Comparison between groups (Mothers).

	Baseline (T0)			Post-Intervention (T1)				2-Month Follow-Up (T2)				Group × Time	
	Intervention (n = 61)			Intervention (n = 52)				Intervention (n = 42)				Intervention (n = 42)	
	Control (n = 51)			Control (n = 43)				Control (n = 39)				Control (n = 39)	
	*M*	*SD*	*p* ^a^	*M*	*SD*	*F*	*p* ^b^	*M*	*SD*	*F*	*p* ^b^	*F*	*p* ^c^
**Resilience**													
Intervention	3.20	(0.59)	0.27	3.47	(0.57)	14.257	**<0.001**	3.42	(0.64)	10.934	**0.001**	9.552	**<0.001**
Control	3.33	(0.67)		3.26	(0.71)			3.24	(0.62)				
**Emotion regulation**													
Intervention	2.77	(0.67)	0.24	3.03	(0.66)	7.634	**0.007**	3.02	(0.68)	17.905	**<0.001**	10.540	**<0.001**
Control	2.97	(0.75)		2.88	(0.74)			2.80	(0.66)				
**Future outlook**													
Intervention	3.51	(0.84)	0.53	3.92	(0.70)	14.860	**<0.001**	3.92	(0.83)	8.572	**0.004**	7.383	**0.001**
Control	3.58	(0.96)		3.49	(0.98)			3.52	(0.92)				
**Novelty seeking**													
Intervention	3.54	(0.74)	0.47	3.71	(0.75)	3.412	0.068	3.57	(0.84)	0.177	0.676	3.734	**0.028**
Control	3.62	(0.75)		3.59	(0.76)			3.61	(0.70)				
**Trait Anger**													
Intervention	27.83	(5.79)	0.58	24.38	(6.07)	7.153	**0.009**	25.00	(6.22)	2.805	0.098	4.145	**0.019**
Control	26.84	(4.35)		26.05	(4.48)			26.13	(4.97)				
**Anger toward children**													
** Anger Out**													
Intervention	23.12	(4.61)	0.31	19.87	(19.60)	6.942	**0.010**	20.66	(4.50)	4.329	**0.041**	4.697	**0.012**
Control	22.09	(4.12)		22.33	(23.29)			21.54	(4.54)				
** Anger In**													
Intervention	16.10	(2.59)	0.63	16.86	(2.96)	0.101	0.751	16.39	(2.84)	0.644	0.425	0.403	0.669
Control	16.05	(2.34)		17.00	(3.25)			16.74	(3.31)				
** Anger Control**													
Intervention	17.25	(3.08)	0.81	19.63	(2.85)	14.122	**<0.001**	18.98	(3.20)	6.643	**0.012**	3.205	**0.046**
Control	17.19	(2.81)		17.67	(2.82)			17.33	(2.90)				
**Anger toward partner**													
** Anger Out**													
Intervention	21.35	(6.09)	0.47	18.48	(5.23)	8.875	**0.004**	19.62	(5.17)	0.930	0.338	4.326	**0.017**
Control	21.86	(5.09)		21.16	(5.60)			20.56	(5.38)				
** Anger In**													
Intervention	19.23	(3.96)	0.61	18.85	(4.44)	0.536	0.466	18.52	(3.65)	0.000	0.988	0.725	0.488
Control	18.19	(4.04)		18.51	(4.25)			18.03	(4.13)				
** Anger Control**													
Intervention	17.59	(3.98)	0.59	18.80	(3.26)	4.628	**0.034**	18.71	(2.94)	6.193	**0.015**	4.935	**0.016**
Control	17.63	(3.38)		17.60	(3.35)			17.36	(3.21)				
**Self esteem**													
Intervention	30.54	(6.63)	0.42	34.25	(8.10)	7.389	**0.008**	35.57	(6.84)	18.767	**<0.001**	9.788	**<0.001**
Control	31.86	(7.81)		32.07	(7.36)			31.44	(7.92)				
**Family function**													
Intervention	6.00	(2.74)	0.77	7.46	(2.51)	7.968	**0.006**	7.52	(2.61)	3.996	**0.049**	3.679	**0.030**
Control	5.84	(2.75)		6.47	(2.47)			6.41	(2.58)				
**Cognition toward child’s misbehavior**													
** Hostile attribution**													
Intervention	1.95	(0.75)	0.06	1.76	(0.76)	2.853	0.095	1.71	(0.74)	6.945	**0.010**	2.449	0.093
Control	2.12	(0.82)		2.08	(0.83)			2.14	(0.85)				
** Negative attribution**													
Intervention	3.22	(0.99)	0.83	2.68	(1.07)	2.708	0.103	2.66	(1.11)	7.121	**0.009**	3.828	**0.026**
Control	3.14	(0.83)		2.92	(0.92)			3.07	(0.84)				
** Positive attribution**													
Intervention	3.71	(0.64)	0.30	4.10	(0.53)	16.636	**<0.001**	4.01	(0.66)	9.227	**0.003**	5.066	**0.009**
Control	3.61	(0.72)		3.68	(0.66)			3.58	(0.73)				
**Parenting emotion**													
** Burden from parenting**													
Intervention	10.85	(2.24)	0.65	9.52	(2.44)	8.956	**0.004**	9.36	(2.48)	2.626	0.109	6.770	**0.002**
Control	10.26	(2.55)		10.45	(2.49)			9.85	(2.88)				
** Burden from child’s attitude/behavior**													
Intervention	13.37	(2.98)	0.87	11.72	(3.10)	22.098	**<0.001**	11.98	(3.35)	9.237	**0.003**	14.008	**<0.001**
Control	13.23	(2.79)		13.85	(3.08)			13.21	(3.51)				
** Anxiety about parenting**												.	
Intervention	12.42	(2.35)	0.58	10.80	(3.11)	11.606	**0.001**	10.36	(2.76)	3.355	0.071	7.890	**<0.001**
Control	12.14	(2.35)		12.34	(2.82)			11.10	(3.24)				
** Anxiety about child’s development**													
Intervention	9.10	(3.20)	0.83	7.86	(3.34)	7.567	**0.007**	7.88	(2.77)	6.346	**0.014**	5.027	**0.009**
Control	9.14	(3.39)		9.31	(3.61)			9.28	(3.87)				
** Positive emotion about parenting**													
Intervention	14.29	(1.86)	0.08	14.56	(1.80)	4.413	**0.038**	14.69	(1.63)	5.541	**0.021**	4.015	**0.022**
Control	13.77	(1.96)		13.69	(1.88)			13.15	(3.13)				
**Marital communication attitude**													
** Compassion toward partner**													
Intervention	2.90	(0.56)	0.58	3.11	(0.54)	4.060	**0.047**	3.07	(0.53)	3.509	0.065	2.214	0.116
Control	2.83	(0.56)		2.92	(0.50)			2.91	(0.52)				
** Compassion from partner**													
Intervention	2.74	(0.73)	0.97	2.95	(0.77)	3.733	0.056	3.06	(0.66)	5.389	**0.023**	2.424	0.095
Control	2.75	(0.76)		2.78	(0.63)			2.78	(0.66)				
**Problem focused coping**													
** Solution seeking**													
Intervention	3.62	(0.76)	0.54	4.01	(0.54)	15.638	**<0.001**	3.75	(0.72)	6.061	**0.016**	5.426	**0.006**
Control	3.71	(0.74)		3.57	(0.72)			3.43	(0.79)				
** Solution behavior**													
Intervention	3.55	(0.76)	0.53	3.88	(0.58)	11.918	**0.001**	3.77	(0.81)	6.435	**0.013**	6.731	**0.002**
Control	3.71	(0.75)		3.49	(0.82)			3.41	(0.93)				

^a^: None of the differences between groups was statistically significant by Student’s *t*-test. ^b^: ANCOVA adjusted for the baseline at each measurement time. Statistically significant numbers are bolded. ^c^: Repeated measures ANOVA for the group effect over the study period. Statistically significant numbers are bolded. ANCOVA: Analysis of Covariance, ANOVA: Analysis of Variance, *M*: Mean, *SD*: Standard deviation.

**Table 4:** Comparison between groups (Fathers).

	Baseline (T0)			Post-Intervention (T1)				2-Month Follow-Up (T2)				Group × Time	
	Intervention (n = 52)			Intervention (n = 39)				Intervention (n = 24)				Intervention (n = 24)	
	Control (n = 36)			Control (n = 27)				Control (n = 21)				Control (n = 21)	
	*M*	*SD*	*p* ^a^	*M*	*SD*	*F*	*p* ^b^	*M*	*SD*	*F*	*p* ^b^	*F*	*p* ^c^
**Resilience**													
Intervention	3.58	(0.47)	0.96	3.53	(0.44)	0.159	0.692	3.43	(0.46)	0.313	0.579	0.025	0.975
Control	3.59	(0.51)		3.52	(0.56)			3.53	(0.39)				
**Emotion regulation**													
Intervention	3.55	(0.56)	0.91	3.48	(0.43)	0.113	0.738	3.37	(0.48)	0.909	0.346	0.285	0.753
Control	3.57	(0.64)		3.44	(0.75)			3.48	(0.49)				
**Future outlook**													
Intervention	3.65	(0.76)	0.81	3.54	(0.64)	1.256	0.267	3.49	(0.76)	0.150	0.701	0.055	0.946
Control	3.62	(0.70)		3.66	(0.78)			3.63	(0.71)				
**Novelty seeking**													
Intervention	3.58	(0.62)	0.89	3.57	(0.67)	0.552	0.460	3.48	(0.62)	0.341	0.563	0.425	0.657
Control	3.60	(0.60)		3.51	(0.56)			3.53	(0.54)				
**Trait Anger**													
Intervention	23.35	(4.41)	0.91	22.74	(4.08)	0.011	0.917	23.75	(4.29)	0.020	0.887	0.092	0.912
Control	23.22	(5.68)		22.89	(5.87)			23.71	(5.20)				
**Anger toward children**													
** Anger Out**													
Intervention	16.85	(4.03)	0.68	17.64	(13.37)	1.078	0.303	17.79	(3.28)	0.043	0.836	0.406	0.669
Control	17.25	(4.84)		18.69	(20.09)			18.38	(4.38)				
** Anger In**													
Intervention	15.37	(2.28)	0.03	16.46	(2.61)	2.272	0.137	16.79	(2.69)	0.040	0.842	1.122	0.335
Control	16.58	(2.70)		16.30	(2.60)			16.71	(2.57)				
** Anger Control**													
Intervention	19.25	(2.76)	0.27	19.59	(2.49)	0.005	0.944	19.75	(2.13)	0.031	0.860	1.135	0.331
Control	20.00	(3.36)		20.15	(3.70)			20.43	(3.20)				
**Anger toward partner**													
** Anger Out**													
Intervention	19.27	(3.99)	0.75	18.47	(3.90)	0.166	0.685	18.71	(4.33)	0.778	0.383	0.454	0.638
Control	19.58	(4.75)		19.26	(5.79)			19.86	(4.84)				
** Anger In**													
Intervention	17.58	(4.18)	0.17	18.13	(3.37)	1.044	0.311	18.67	(4.49)	0.003	0.954	0.787	0.462
Control	18.82	(4.03)		17.64	(3.47)			18.89	(4.32)				
** Anger Control**													
Intervention	19.60	(3.21)	0.62	19.89	(2.47)	0.306	0.582	19.58	(2.36)	0.559	0.459	1.165	0.322
Control	19.94	(3.29)		19.15	(3.82)			19.14	(3.57)				
**Self esteem**													
Intervention	34.58	(5.91)	0.63	34.10	(4.50)	0.013	0.911	33.79	(5.82)	0.636	0.430	0.542	0.586
Control	35.17	(5.56)		34.81	(6.93)			34.24	(5.81)				
**Family function**													
Intervention	6.68	(2.44)	0.34	6.87	(2.68)	0.002	0.964	6.52	(2.79)	0.045	0.832	0.276	0.761
Control	7.23	(2.20)		7.35	(2.28)			6.90	(2.72)				
**Cognition toward child’s misbehavior**													
** Hostile attribution**													
Intervention	1.67	(0.58)	0.78	1.74	(0.60)	0.000	0.992	1.71	(0.71)	2.102	0.155	1.184	0.316
Control	1.70	(0.58)		1.74	(0.51)			1.93	(0.64)				
** Negative attribution**													
Intervention	2.30	(0.85)	0.09	2.38	(0.77)	0.799	0.375	2.53	(0.84)	0.181	0.673	1.254	0.296
Control	2.63	(0.90)		2.71	(0.88)			2.57	(0.60)				
** Positive attribution**													
Intervention	3.81	(0.53)	0.40	3.79	(0.49)	0.160	0.691	3.81	(0.46)	0.517	0.476	1.239	0.300
Control	3.71	(0.57)		3.80	(0.56)			3.85	(0.58)				
**Parenting emotion**													
** Burden from parenting**													
Intervention	7.96	(2.01)	0.92	8.65	(2.02)	1.876	0.218	8.55	(1.99)	1.028	0.317	1.358	0.216
Control	7.92	(1.89)		7.72	(1.90)			7.81	(1.78)				
** Burden from child’s attitude/behavior**													
Intervention	9.96	(2.29)	0.39	10.54	(2.43)	0.150	0.878	10.23	(2.37)	0.006	0.936	0.108	0.854
Control	10.44	(2.75)		10.76	(2.12)			10.24	(2.32)				
** Anxiety about parenting**													
Intervention	9.80	(2.53)	0.93	9.99	(2.54)	0.676	0.764	9.95	(2.50)	0.535	0.469	0.578	0.560
Control	9.74	(2.63)		10.54	(2.19)			10.14	(2.35)				
** Anxiety about child’s development**													
Intervention	7.94	(2.83)	0.05	8.99	(2.75)	1.652	0.341	8.82	(2.81)	1.364	0.250	1.467	0.249
Control	9.22	(3.03)		10.02	(2.25)			10.00	(2.47)				
** Positive emotion about parenting**													
Intervention	14.33	(1.49)	0.34	13.98	(1.65)	0.308	0.675	13.18	(1.76)	0.107	0.746	0.215	0.689
Control	13.94	(2.01)		13.14	(1.90)			12.95	(1.80)				
**Marital communication attitude**													
** Compassion toward partner**													
Intervention	2.94	(0.48)	0.65	2.91	(0.44)	0.163	0.688	2.93	(0.42)	0.000	0.984	0.054	0.947
Control	2.90	(0.39)		2.95	(0.38)			2.90	(0.30)				
** Compassion from partner**													
Intervention	2.97	(0.69)	0.69	2.88	(0.65)	0.098	0.755	2.85	(0.61)	0.802	0.376	1.043	0.361
Control	2.92	(0.52)		2.87	(0.50)			2.68	(0.65)				
**Problem focused coping**													
** Solution seeking**													
Intervention	3.48	(0.80)	0.79	3.29	(0.78)	1.423	0.237	3.34	(0.67)	0.760	0.065	1.942	0.156
Control	3.53	(0.85)		3.47	(0.89)			3.65	(0.50)				
** Solution behavior**													
Intervention	3.50	(0.75)	0.49	3.34	(0.76)	0.840	0.363	3.15	(0.66)	0.056	0.730	2.881	0.067
Control	3.38	(0.84)		3.43	(0.92)			3.56	(0.62)				

^a^: None of the differences between groups was statistically significant by Student’s *t*-test. ^b^: ANCOVA adjusted for the baseline at each measurement time. ^c^: Repeated measures ANOVA for the group effect over the study period. ANCOVA: Analysis of Covariance, ANOVA: Analysis of Variance, *M*: Mean, *SD*: Standard deviation.

## Data Availability

The data are not publicly available. Please contact the corresponding author with any requests for information.
